# Screen Exposure during Early Life and the Increased Risk of Astigmatism among Preschool Children: Findings from Longhua Child Cohort Study

**DOI:** 10.3390/ijerph17072216

**Published:** 2020-03-26

**Authors:** Lihua Huang, Gui-You Yang, Katrina L. Schmid, Jing-Yi Chen, Chen-Guang Li, Guan-Hao He, Zeng-Liang Ruan, Wei-Qing Chen

**Affiliations:** 1 Department of Medical Statistics and Epidemiology, Guangzhou Key Laboratory of Environmental Pollution and Health Assessment, Guangdong Provincial Key Laboratory of Food, Nutrition and Health, School of Public Health, Sun Yat-sen University, Guangzhou 510080, China; hlihua2@mail2.sysu.edu.cn (L.H.); yanggy7@mail2.sysu.edu.cn (G.-Y.Y.); chenjy246@mail2.sysu.edu.cn (J.-Y.C.); lichg3@mail2.sysu.edu.cn (C.-G.L.); heguanh@mail2.sysu.edu.cn (G.-H.H.); ruanzliang@mail2.sysu.edu.cn (Z.-L.R.); 2 School of Optometry and Vision Science, Faculty of Health, Queensland University of Technology, Brisbane, 4059 QLD, Australia; k.schmid@qut.edu.au; 3 Department of Information Management, Xinhua College of Sun Yat-sen University, Guangzhou 510080, China

**Keywords:** screen exposure, astigmatism, preschool children, early life, cross-sectional study

## Abstract

Screen media usage has become increasingly prevalent in daily life with children being exposed to screens at an early age. This is a growing public health concern with evidence linking screen exposure to detrimental health outcomes, whereas relationship between screen exposure and the presence of astigmatism among preschoolers remains unknown, thus we aimed to resolve this issue. During the 2017 survey of the Longhua Child Cohort Study, data of 29,595 preschoolers were collected via a caregiver-reported questionnaire regarding socio-demographics, screen exposure and refraction. Cox regression models were adopted to generate adjusted prevalence ratios (APR) and 95% confidence intervals (CI) to estimate the association between early screen exposure and astigmatism. 28,029 preschoolers were included in the final analysis. After adjustment for potential confounders, screen exposure during early life was significantly associated with the increased risk of astigmatism (APR and 95% CI: 2.25, 1.76–2.88), and the greatest risk was observed in the period from birth to 1-year (APR and 95% CI: 3.10, 2.41–3.98). The risk of astigmatism increased with both the total years of exposure and the average daily duration of screen exposure. Our findings suggested that preschoolers who were exposed to screens during early life might have an increased risk of astigmatism.

## 1. Introduction

Astigmatism is a refractive condition where light rays are not imaged at a single retinal point. It is due to either the cornea or crystalline lens having surface curvatures that are not spherical but rather differ in orthogonal meridians; it can be optically corrected using a cylindrical lens. Astigmatism is relatively common and can have detrimental visual effects if of high levels (>4.00 diopter cylinder) from a young age [1]. It is associated with high myopia [2], and if it occurs early in life can lead to the development of amblyopia; the visual impacts are greater in a developing visual system [3,4]. It is well-known that visual function rapidly develops and improves after birth to 6 years of age, and that the growth of the eye is greatest during the first year of life and slows with age [5,6]. Thus, determination of modifiable risk factors that are present during this period and might be linked to the development of astigmatism, is important. 

With the rapid development of information technology, electronic media devices (e.g., televisions (TVs), computers, smartphones and tablets) are embedded in people’s daily lives for work, education and entertainment [7,8]. Even more dramatic has been the great increase in the use of such devices by young and very young children. A nationally representative survey of the United States found that 68% of children under the age of 2 used screen-based devices during a typical day [7], and another recent survey from Italy showed that 80% of children aged 3 to 5 years used their parent’s smartphone [9]. Excessive screen time is associated with poor sleep, obesity and psychological problems among school-aged children and thus generally considered to be detrimental to children’s health [8]. There are also adverse ocular impacts with several studies reporting that prolonged screen time is linked to asthenopia [10], myopia [11], and low vision [12]. Whether there is any relationship between screen exposure in early life and astigmatism amongst preschoolers is unknown, as is whether any effect is dose and/or age dependent. Due to the increased use of small hand held mobile devices [13], understanding the implications of exposure to both fixed and hand held devices is needed.

The routine vision screening of kindergarten children in Longhua District of Shenzhen and the survey of Longhua Children Cohort Study (LCCS) provided us an opportunity to evaluate the relationship between screen exposure during early life with astigmatism based on a life-course approach [14]. Given that parental history of refractive errors was associated with astigmatism [15,16], we also assessed the combined influence of parental eyesight and screen exposure on astigmatism among preschool children aged 2–7 years in Longhua District, Shenzhen, China.

## 2. Materials and Methods 

### 2.1. Study Participants

All participants were recruited from the LCCS which examined the influence of environmental factors surrounding children’s early life on child psycho-behavioral development; it commenced in September 2014 with once per year follow up [17,18,19,20]. Children were enrolled in the LCCS at the commencement of kindergarten and their primary caregivers completed a self-administered structured questionnaire. In 2017 a total of 29,595 caregivers of children aged 2 to 7 years were approached to complete the screen exposure and vision survey. The study was conducted in accordance with the Declaration of Helsinki, and the protocol was approved by the Ethics Committee of the School of Public Health at Sun Yat-sen University (ethics clearance No. 2015–016). Signed written informed consent was obtained from the primary caregivers. In the present study, children who had missing information on either the socio-demographic characteristics or refractive condition and children who had been diagnosed with another type of refractive error (i.e., did not have normal vision or astigmatism) were excluded. Figure S1 presents the flow diagram of participant selection. Data of 28,029 (95 %) preschoolers were included in the final analysis. 

### 2.2. Data Collection

Primary caregivers completed a self-administered structured questionnaire regarding the parental socio-demographic characteristics including family income, parental age at childbirth, education level and eyesight status (i.e., poor uncorrected vision due to refractive errors versus good uncorrected vision), as well as children’s general information including gender, date of birth, single child or not, birth history (i.e., full-term versus preterm), birth weight, passive smoking, screen exposure during different stages of early life and refractive status (based on the kindergarten vision screening). 

### 2.3. Measurements of Screen Exposure

A set of questions was asked about the screen exposure. Table 1 presents the questions and options regarding screen exposure from birth to 1 year of age. The questions and options shown in Table 1 were repeated for yearly age bands up to 6 years of age (i.e., 1–2 y, 2–3 y, 3–4 y, 4–5 y and 5–6 y). For children aged less than 6 years, screen exposure across the child’s entire life was investigated, while for children aged over 6 years, only exposure from birth to 6 years was assessed. The following variables were used to describe the screen exposure: (1) the child’s age at first exposure, (2) the total years of exposure, and (3) average daily exposure duration. 

### 2.4. Determination of the Presence of Astigmatism 

Since 2017, the vision screening for preschool children is performed by the ophthalmologists from the Longhua District Maternal and Child Health Hospital twice per school year (i.e., spring term and autumn term) in kindergartens. The results of the vision screening are provided to children’s caregivers by teachers, and if the results of screening implied an abnormal refraction (i.e., myopia, astigmatism, hyperopia and other visual abnormalities), parents will be advised to take the child for a more comprehensive eye examination at the ophthalmic clinic of the hospital. At the clinic, the ophthalmologists would suggest the child to receive a comprehensive eye examination and take appropriate measures (e.g., having the child wear glasses to correct vision, etc.) according to the cycloplegic refraction. The ophthalmologists from Longhua District Maternal and Child Health Hospital defined the children’s refractive problem being astigmatism if the measured astigmatism was 1.75 diopter cylinder or more, myopia if the refraction was 0.50 diopter or lower, and hyperopia if the refraction was 2.00 diopter or more for preschool children.

In the present study, therefore, the following questions [21] were asked about the child’s eye conditions: (1) Has your child ever been diagnosed as having poor sight by the oculist? (0 = ‘no’, 1 = ‘yes’, 2 = ‘uncertain’); and if yes, the subsequent questions were asked separately, i.e., questions (2)–(7): Has your child ever been diagnosed as having astigmatism/myopia/hyperopia/strabismus/amblyopia/other common visual problems? (0 = ‘no’, 1 = ‘yes’, 2 = ‘uncertain’). (8) When was your child diagnosed as having the aforementioned poor eyesight? In this study, only children with astigmatism and children without poor sight were included in the analysis. 

### 2.5. Potential Confounders 

According to the previous literature [22,23,24], factors such as a child’s age, gender, premature birth (<37 weeks), birth weight (low birth weight (<2500 g), normal birth weight (2500–4000 g), macrosomia (>4000 g)), parental age at childbirth, parental education level (junior high school and below, high school or technical secondary school, junior college, and graduate and above), monthly household income (<5000, 5000~10000, 10000~15000, 15000~20000, and ≥20000 RMB per month) and passive smoking can impact a child’s refraction and were thus included as potential confounders in this study. Moreover, parental eyesight status (i.e., poor uncorrected vision due to refractive errors versus good uncorrected vision) was the stratification variable in the stratified analysis. 

### 2.6. Statistical Analysis

Means and standard deviations (SD) were used to describe continuous variables, and absolute frequencies and proportions were used for categorical variables. Chi-square tests or t-tests were applied to compare the socio-demographic characteristics and screen exposure between preschoolers with astigmatism and preschoolers with normal eyesight. 

Cox regression modelling [25] was adopted to examine the associations between screen exposure and age of first screen exposure and astigmatism, and to evaluate dose-response relationships of total years of exposure and daily duration of exposure with astigmatism. A crossover analysis, based on different permutations of exposure (Yes) versus no exposure (No) in each year from birth to 3 years old, was performed to explore the sensitive period [14]. To determine the impact of screen exposure on astigmatism in children without a hereditary predisposition towards astigmatism and the combined impact of parental eyesight and screen exposure on astigmatism, the participants were stratified based on the status of parental eyesight (i.e., poor uncorrected vision due to refractive errors versus good uncorrected vision), and the aforementioned analyses were repeated. Also, interactive analyses were performed to examine the interaction of screen exposure (Yes/No) and status of parental eyesight (i.e., poor uncorrected vision due to refractive errors versus good uncorrected vision) on children’s astigmatism. All the multiple regression models above were performed with adjustment for the aforementioned potential confounders, and adjusted prevalence ratio (APR) and 95% confidence intervals (95% CI) were determined [25]. 

Additionally, given that preterm birth has a higher risk of astigmatism than full-term birth [26], stratification analysis was applied to assess the association between screen exposure and astigmatism in two subgroups of preschoolers (i.e., preterm birth and full-term birth). The adjustment for potential confounders including children’s age, gender, maternal age at childbirth, paternal age at childbirth, maternal education level, paternal education level, monthly household income and smoking was also made.

Statistical analyses were performed with R statistical software (version 3.4.0, http://www.r-project.org) and SPSS software (version 23.0; SPSS Inc., Chicago, IL, USA). Two-sided *p* < 0.05 was considered as the significant difference. 

## 3. Results

### 3.1. Characteristics of Preschoolers with Astigmatism and Those with Normal Eyesight

Children who had astigmatism were older (4.7 ± 0.9 vs. 4.6 ± 0.9 years old) and had a larger proportion of preterm birth (10.0% vs. 7.3%) and low birth weight (5.0% vs. 3.2%) compared to children with normal eyesight (Table 2). Other characteristics including child’s gender, parental education, age at childbirth, family income and passive smoking were similar between the two groups. 

### 3.2. Associations between the Initial Age of Screen Exposure and Preschool Astigmatism

**Figure 1:**
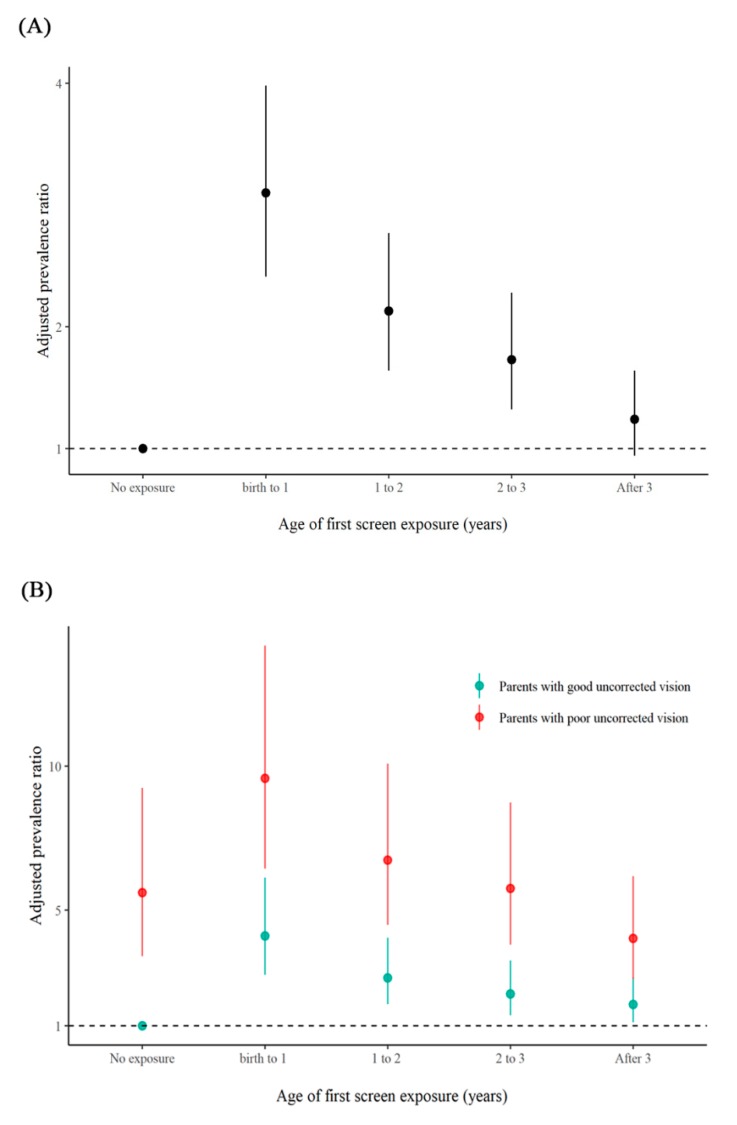
Associations between the initial age of screen exposure and preschool astigmatism. (**A**): Associations between the age of first screen exposure and preschool astigmatism. (**B**): Associations between the age of first screen exposure and preschool astigmatism stratified by the status of parental eyesight.

Compared with those who had no screen exposure, preschoolers exposed to screens had a higher risk of astigmatism (APR: 2.25, 95% CI: 1.76–2.88), and preschoolers whose parents had poor uncorrected vision had an even greater risk of astigmatism than those whose parents had good uncorrected vision (APR: 7.15, 95% CI: 4.84–10.56) (Table S1). What is more, preschoolers who were initially exposed to screens during the first three years of life had statistically significant higher risk of astigmatism, and those who were initially exposed to screens in the first year of life had the highest risk (Figure 1A). Furthermore, stratified analyses based on parental eyesight status showed similar trends (Figure 1B). Additionally, the interaction of screen exposure and status of parental eyesight on preschool astigmatism was statistically significant (*p* < 0.05).

### 3.3. Relationships of Average Daily Exposure Duration and the Total Years of Exposure with Preschool Astigmatism

Compared with no screen exposure, average daily screen time over 120 min had the strongest relationship to astigmatism risk, and the strength of the association increased as the number of total years of exposure (i.e., 1, 2, 3, 4, ≥5 years) increased (APR and 95% CI for 1: 2.10, 1.04–4.22; for 2: 1.70, 1.05–2.77; for 3: 2.34, 1.68–3.26, for 4: 2.54, 1.87–3.45; and for ≥5: 3.65, 2.75–4.84) (Table 3). What is more, the association between the total years of screen exposure and preschool astigmatism became weaker as average daily screen time (i.e., >120 min, 60–120 min and <60 min) decreased (Table 3). A similar trend was observed in the results of the stratified analyses based on the status of parental eyesight (Table 3).

### 3.4. Associations between Exposure to Screens during Postnatal Three Years and Astigmatism 

Compared with preschoolers never exposed to screens during their first three years of life, all subgroups except the one only exposed in the second year of life had statistically significant higher risk of astigmatism, and the subgroup exposed to screens during the entire three years had the highest risk of astigmatism (APR: 2.78, 95% CI: 2.42–3.19) (Table 4). What’s more, children who were exposed to screens only in the first year of life had a higher risk of astigmatism than children exposed to screens only in the postnatal second or third year of life (APR and 95% CI: for subgroup exposed to screens only in the postnatal first year: 1.91, 1.44–2.54; for subgroup exposed to screens only in the postnatal second year: 1.08, 0.70–1.69; for subgroup exposed to screens only in the postnatal third year: 1.48, 1.25–1.75) (Table 4). A similar trend was observed in the results of the stratified analyses based on the status of parental eyesight (Table 4).

### 3.5. Sensitivity Analysis 

The associations between screen exposure (Yes/No), the initial age of screen exposure and astigmatism were still significant in both subgroups (i.e., preterm birth and full-term birth), and preterm preschoolers had a greater risk of astigmatism than those born at term (Table S2). 

## 4. Discussion

It was observed that preschoolers exposed to screens in early life had a high risk of astigmatism and the risk increased as the daily duration and total years of exposure increased. As the age of first screen exposure delayed, the strength of association reduced. Association between screen exposure and astigmatism was still significant in preschoolers whose parents had good uncorrected vision, and strength of the associations was enhanced in preschoolers whose parents had poor uncorrected vision, indicative of the combined impact of parental history of poor vision and screen exposure on astigmatism. This study supports the need to promote public health awareness of the visual harm of screen exposure during early life, especially for those parents who have poor uncorrected vision.

Screen exposure may have adverse health effects, particularly on eyes. Studies regarding the impact of excessive screen time on ocular-related health problems, have been largely confined to the ocular symptoms related to eye fatigue, myopia and low vision [10,11,27]. Here we found that screen exposure was associated with a higher risk of astigmatism amongst preschoolers and the risk increased as the daily duration and total years of exposure increased. Since limited research has been undertaken about screen exposure and astigmatism, no other studies are available for direct comparison. However, a number of related studies support our findings. Several cross-sectional studies in older children aged 6 to 18 years show a positive association between poor vision and excessive time on TV viewing and Internet use (more than 3 hours/day), and another study in a similar age group, 7–15 years, suggested that regular use of computers is a potential risk factor for refractive error [12,28,29,30]. Inspired by life-course epidemiology [14], we found that exposure to screens during the first year of life had the greater impact on preschool astigmatism than that during the second year and the third year, which indicated that the period from birth to 1-year-old was the sensitive period of their association. Intriguingly, such finding supports the idea that environmental impacts during the critical period of visual system development have the most effect [5,6], which possibly revealed the important timing of interventions to prevent astigmatism among preschoolers. However, as cross-sectional studies can only determine associations, longitudinal studies are required to prove if exposure to screens causes high prevalence of astigmatism.

Previous studies have showed that children whose parents have myopia and/or astigmatism are at higher risk of astigmatism than those whose parents have good vision [15,16]. Analogous to this, astigmatism was more prevalent in children whose parents had poor uncorrected vision than in those whose parents had good uncorrected vision, regardless of the screen exposure status. Furthermore, compared with preschoolers whose parents had good uncorrected vision, the strength of the associations between screen exposure and astigmatism was stronger in preschoolers whose parents had poor uncorrected vision, suggesting a combined impact of genetic predisposition and screen exposure on astigmatism. However, that children and their parents share similar environments, nutrition, culture and education may also further strengthen this relationship [31]. Further studies with susceptibility gene measurement are needed to clarify the effect of heredity on astigmatism. 

The physiological mechanism via which screen exposure and astigmatism are linked is unknown. There are several untested hypotheses. Excessive screen time at close distances might cause excessive accommodation, thus leading to overworking of the ciliary muscles of the eyes of children and impacting the natural development of the crystalline lens, impacting its curvature [32]. Alternately, looking at the screens up close may lead to changes in the shape of the children’s corneas, due to variations in the palpebral aperture and eye movements performed during the tasks [33], or the mechanical interactions between the cornea and the eyelids [1]. Also, it may be that staring down at the screen increases pressure from eyelids on the cornea, which can result in increased corneal astigmatism [34]. Future ocular biometry studies in very young children and information about posture adopted while they look at handheld screens could provide insight into the underlying mechanism.

This study is one of the very few studies investigating the association between screen exposure during the early life and preschool astigmatism. However, it has several limitations. The astigmatism cases were identified as positive in a large sample screening and further diagnosed as astigmatism at the ophthalmic clinic, and reported by parents or other caregivers, which might lead to information bias. The findings from our published study [21], which used the same measurement of astigmatism, were consistent with the findings from the multi-ethnic pediatric eye disease and Baltimore pediatric eye disease studies [23] which used cycloplegic refraction to define astigmatism, thus illustrating that to some extent, our measurements were reliable. Additionally, the measurement of outcome was monotonous and not graded. Since a previous study from Singapore suggested that playing video games and computers may be associated with astigmatism severity [15], it would be more interesting and insightful if further studies could include the distribution of different levels of astigmatism and its association with screen exposure. Information on screen exposure from birth to 6 years of age was recalled and relied on parental or other caregivers’ reporting, which might lead to information bias. What’s more, information regarding screen exposure in kindergarten might not be fully reported, which could lead to an underestimation of screen exposure. In the present electronic information age, measurements of screen exposure are warranted to be developed. Some characteristics of screen exposure that may have a unique effect on or a different mechanism in affecting astigmatism, such as posture when children are exposed to screens, the screen size and resolution of screen devices, pattern of screen use (i.e., intermittent use, continuous use), were not investigated in the present study. Future studies should take these details regarding screen devices into consideration. Although the data was corrected for age, gender, premature birth, birth weight, parental education, age at childbirth, family income and passive smoking, there may be other unmeasured confounders like other types of near work (e.g., writing, reading) which might impact the findings. Finally, the nature of cross-sectional study design could only provide suggestive but not confirmative causality regarding the association between screen exposure and preschool astigmatism. Further longitudinal studies with objective measurements should be conducted to investigate the cause-effect relationship.

## 5. Conclusions

Screen exposure in early life could be associated with a higher risk of astigmatism and the postnatal first year might be the sensitive period for their associations. However, considering the poor assessment of astigmatism in our study, it’s premature to conclude that early screen time leads to astigmatism with current data. Further longitudinal studies including objective measurements performed with cycloplegia are needed to verify our finding. 

## Figures and Tables

**Table 1:** Questions and options regarding screen exposure from birth to 1 year of age.

No.	Questions	Options
Q1.	Was your child involved in watching TV or computers or laptops from birth to 1 year of age?	0 = “no” 1 = “yes”
Q1.1	If “yes”, how long on average per day was he/she exposed to these screen devices?	1 = “<30 min”, 2 = “30–60 min”, 3 = “60–90 min”, 4 = “90–120 min”, 5 = “>120 min”
Q2.	Was your child involved in using smartphones, tablets, and other handheld electronic screens from birth to 1 year of age?	0 = “no” 1 = “yes”
Q2.1	If “yes”, how long on average per day was he/she exposed to these electronic screens?	1 = “<30 min”, 2 = “30–60 min”, 3 = “60–90 min”, 4 = “90–120 min”, 5 = “>120 min”

**Table 2:** Characteristics and comparison of preschoolers with astigmatism and those with normal eyesight.

Characteristic	Total (N = 28,029)	Normal Eyesight (N = 26,007)	Astigmatism (N = 2022)	*p*
Age (years)	4.6 (0.9)	4.6 (0.9)	4.7 (0.9)	0.001
Gender				
male	15,208 (54.3)	14,088 (54.2)	1120 (55.4)	0.299
female	12,821 (45.7)	11,919 (45.8)	902 (44.6)	
Maternal Education				0.950
Junior high school and below	7006 (25.0)	6491 (25.0)	515 (25.5)	
High school or technical secondary school	8237 (29.4)	7650 (29.4)	587 (29.0)	
Junior college	6859 (24.5)	6362 (24.5)	497 (24.6)	
Graduate and above	5927 (21.1)	5504 (21.2)	423 (20.9)	
Paternal Education				0.465
Junior high school and below	5787 (20.6)	5369 (20.6)	418 (20.7)	
High school or technical secondary school	7600 (27.1)	7040 (27.1)	560 (27.7)	
Junior college	6428 (22.9)	5947 (22.9)	481 (23.8)	
Graduate and above	8214 (29.3)	7651 (29.4)	563 (27.8)	
Monthly household income				0.139
<5000	4124 (14.7)	3823 (14.7)	301 (14.9)	
5000~10,000	7385 (26.3)	6813 (26.2)	572 (28.3)	
10,000~15,000	5364 (19.1)	4974 (19.1)	390 (19.3)	
15,000~20,000	3899 (13.9)	3621 (13.9)	278 (13.7)	
≥20,000	7257 (25.9)	6776 (26.1)	481 (23.8)	
Maternal age at pregnancy	27.14 (4.23)	27.15 (4.23)	27.09 (4.17)	0.600
Paternal age at pregnancy	29.71 (4.81)	29.71 (4.81)	29.63 (4.85)	0.473
Birth weight				<0.001
Low birth weight (<2500 g)	24,157 (86.2)	22,445 (86.3)	1712 (84.7)	
Normal birth weight (2500–4000 g)	945 (3.4)	844 (3.2)	101 (5.0)	
Macrosomia (>4000 g)	2927 (10.4)	2718 (10.5)	209 (10.3)	
Preterm birth				<0.001
No	25,927 (92.5)	24,107 (92.7)	1820 (90.0)	
Yes	2102 (7.5)	1900 (7.3)	202 (10.0)	
Passive smoking				<0.001
No	17,583 (62.7)	16,442 (63.2)	1141 (56.4)	
Yes	10,446 (37.3)	9565 (36.8)	881 (43.6)	
Parental eyesight status				<0.001
Good uncorrected vision	16,116 (57.5)	15,366 (59.1)	750 (37.1)	
Poor uncorrected vision due to refractive errors	11,913 (42.5)	10,641 (40.9)	1272 (62.9)	

**Table 3:** Associations between the total years of exposure and preschool astigmatism grouped by average daily screen time.

Exposure to Screen		Case/Total	Prevalence (%)	APR ^#^ (95% CI)	Parents with Good Uncorrected Vision			Parents with Poor Uncorrected Vision		
Average Time (min/day)	Cumulative Years				Case/Total	Prevalence (%)	APR ^#^ (95% CI)	Case/Total	Prevalence (%)	APR ^#^ (95% CI)
0	0	65/1972	3.3	1.0	26/1496	1.7	1.00	39/476	8.2	5.51 (3.35, 9.07) ***
<60										
	1	82/1487	5.5	1.67 (1.21, 2.32) **	30/977	3.1	1.81 (1.07, 3.07) *	52/510	10.2	6.94 (4.32, 11.15) ***
	2	109/2066	5.3	1.59 (1.17, 2.16) **	39/1240	3.2	1.85 (1.13, 3.05) *	70/826	8.5	5.75 (3.66, 9.04) ***
	3	145/2382	6.1	1.86 (1.39, 2.49) ***	55/1341	4.1	2.43 (1.52, 3.88) ***	90/1041	8.7	5.94 (3.83, 9.22) ***
	4	144/1699	8.5	2.59 (1.93, 3.47) ***	49/908	5.4	3.17 (1.97, 5.10) ***	95/791	12.0	8.07 (5.22, 12.49) ***
	≥5	114/1228	9.3	2.90 (2.13, 3.94) ***	39/698	5.6	3.28 (1.99, 5.39) ***	75/530	14.2	9.62 (6.14, 15.08) ***
60–120										
	1	18/501	3.6	1.07 (0.63, 1.81)	10/295	3.4	2.02 (0.97, 4.20)	8/206	3.9	2.59 (1.17, 5.74) *
	2	76/1638	4.6	1.36 (0.98, 1.90)	30/957	3.1	1.82 (1.07, 3.07) *	46/681	6.8	4.42 (2.72, 7.17) ***
	3	200/3156	6.3	1.92 (1.45, 2.54) ***	67/1768	3.8	2.25 (1.43, 3.54) ***	133/1388	9.6	6.31 (4.14, 9.64) ***
	4	285/3521	8.1	2.41 (1.84, 3.16) ***	99/1916	5.2	2.96 (1.92, 4.56) ***	186/1605	11.6	7.48 (4.95, 11.31) ***
	≥5	356/3779	9.4	2.95 (2.26, 3.85) ***	139/2068	6.7	3.90 (2.56, 5.95) ***	217/1711	12.7	8.37 (5.55, 12.61) ***
>120										
	1	9/134	6.7	2.10 (1.04, 4.22) *	6/77	7.8	4.92 (2.02, 11.97) ***	3/57	5.3	3.36 (1.02, 11.12) *
	2	22/382	5.8	1.70 (1.05, 2.77) *	7/225	3.1	1.75 (0.76, 4.02)	15/157	9.6	6.35 (3.36, 12.03) ***
	3	77/1002	7.7	2.34 (1.68, 3.26) ***	27/556	4.9	2.91 (1.69, 4.98) ***	50/446	11.2	7.31 (4.54, 11.77) ***
	4	111/1292	8.6	2.54 (1.87, 3.45) ***	48/678	7.1	4.08 (2.53, 6.58) ***	63/614	10.3	6.52 (4.12, 10.31) ***
	≥5	209/1790	11.7	3.65 (2.75, 4.84) ***	79/916	8.6	5.11 (3.27, 7.98) ***	130/874	14.9	9.57 (6.26, 14.63) ***

APR: adjusted prevalence ratio; CI: confidence intervals; ^#^: Adjusted for children’s age, gender, maternal age at childbirth, paternal age at childbirth, maternal education level, paternal education level, monthly household income, preterm birth, birth weight and passive smoking during early childhood; *: *p* < 0.05; **: *p* < 0.01; ***: *p* < 0.001.

**Table 4:** Associations between exposure to screens during early life (postnatal three years) and astigmatism.

Exposure to Screens before 3 Years			Case/Total	Prevalence (%)	APR ^#^ (95% CI)	Parents with Good Uncorrected Vision			Parents with Poor Uncorrected Vision		
birth-1	1–2	2–3				Case/Total	Prevalence (%)	APR ^#^ (95% CI)	Case/Total	Prevalence (%)	APR ^#^ (95% CI)
No	No	No	278/6897	4.0	1.00	118/4512	2.6	1.00	160/2385	6.7	2.88 (2.27, 3.67) ***
Yes	No	No	58/759	7.6	1.91 (1.44, 2.54) ***	22/481	4.6	1.69 (1.07, 2.67) *	36/278	13.0	5.48 (3.77, 7.97) ***
No	Yes	No	21/474	4.4	1.08 (0.70, 1.69)	8/298	2.7	1.00 (0.49, 2.05)	13/176	7.4	2.93 (1.65, 5.20) ***
No	No	Yes	265/4556	5.8	1.48 (1.25, 1.75) ***	91/2549	3.6	1.40 (1.07, 1.84) *	174/2007	8.7	3.81 (3.01, 4.83) ***
Yes	Yes	No	51/545	9.4	2.46 (1.83, 3.32) ***	25/333	7.5	2.95 (1.92, 4.55) ***	26/212	12.3	5.35 (3.49, 8.18) ***
Yes	No	Yes	52/657	7.9	1.99 (1.48, 2.67) ***	22/401	5.5	2.04 (1.29, 3.22) **	30/256	11.7	4.94 (3.31, 7.38) ***
No	Yes	Yes	426/5904	7.2	1.88 (1.62, 2.19) ***	142/3100	4.6	1.85 (1.45, 2.36) ***	284/2803	10.1	4.54 (3.65, 5.64) ***
Yes	Yes	Yes	871/8237	10.6	2.78 (2.42, 3.19) ***	322/4442	7.3	2.86 (2.31, 3.53) ***	549/3797	14.5	6.46 (5.28, 7.90) ***

APR: adjusted prevalence ratio; CI: confidence intervals; ^#^: Adjusted for children’s age, gender, maternal age at childbirth, paternal age at childbirth, maternal education level, paternal education level, monthly household income, preterm birth, birth weight and passive smoking; *: *p* < 0.05; **: *p* < 0.01; ***: *p* < 0.001.

## Supplementary Materials

The following are available online at https://www.mdpi.com/1660-4601/17/7/2216/s1, Figure S1: The flow diagram of participant selection, Table S1: Associations between screen exposure and astigmatism among preschoolers, Table S2: Sensitivity analysis.
