# Supplementary material for: Screen Exposure during Early Life and the Increased Risk of Astigmatism among Preschool Children: Findings from Longhua Child Cohort Study
Source: Int J Environ Res Public Health. 2020 Mar 26;17(7):2216. doi: 10.3390/ijerph17072216 (PMC7177845; doi:10.3390/ijerph17072216)
Supplement: Supplementary file 1 [file ijerph-17-02216-s001.zip › ijerph-750615-supplementary/TableS1-2_25_March_clean version.docx]

**Table S1.** Associations between screen exposure and astigmatism among preschoolers

| **Screen exposure** | **Case/Total** | **Prevalence**  **(%)** | **APR^#^ (95% CI)** | **Parents with poor uncorrected vision** | | | **Parents with poor uncorrected vision** | | |
| --- | --- | --- | --- | --- | --- | --- | --- | --- | --- |
|  |  |  |  | **Case/Total** | **Prevalence**  **(%)** | **APR^#^ (95% CI)** | **Case/Total** | **Prevalence**  **(%)** | **APR^#^ (95% CI)** |
| Screen exposure |  |  |  |  |  |  |  |  |  |
| No | 65/1972 | 3.30 | 1.00 | 1496/26 | 1.74 | 1.00 | 39/476 | 8.19 | 5.69 (3.46, 9.37)^***^ |
| Yes | 1957/26057 | 7.51 | 2.25 (1.76, 2.88)^***^ | 14620/726 | 4.97 | 2.87 (1.94, 4.25)^***^ | 1233/11437 | 10.78 | 7.15 (4.84, 10.56)^***^ |
| Initial age of exposure to screens | | |  |  |  |  |  |  |  |
| No | 65/1972 | 3.30 | 1.00 | 26/1496 | 1.74 | 1.00 | 39/476 | 8.19 | 5.61 (3.41, 9.24)^***^ |
| 0 to 1 year old | 1032/10198 | 10.12 | 3.10 (2.41, 3.98)^***^ | 391/5657 | 6.91 | 4.12 (2.77, 6.13)^***^ | 641/4541 | 14.12 | 9.57 (6.45, 14.18)^***^ |
| 1 to 2 years old | 447/6378 | 7.01 | 2.13 (1.64, 2.77)^***^ | 150/3398 | 4.41 | 2.67 (1.76, 4.06)^***^ | 297/2980 | 9.97 | 6.74 (4.50, 10.08)^***^ |
| 2 to 3 years old | 265/4556 | 5.82 | 1.73 (1.32, 2.28)^***^ | 91/2549 | 3.57 | 2.11 (1.37, 3.27)^**^ | 174/2007 | 8.67 | 5.76 (3.81, 8.73)^***^ |
| After 3 years old | 213/4925 | 4.32 | 1.24 (0.94, 1.64) | 92/3016 | 3.05 | 1.74 (1.13, 2.69)^**^ | 121/1909 | 6.34 | 4.03 (2.63, 6.18)^***^ |

APR: adjusted prevalence ratio; CI: confidence intervals; #: Adjusted for children’s age, gender, maternal age at childbirth, paternal age at childbirth, maternal education level, paternal educational level, monthly household income, preterm birth, birth weight and passive smoking during early childhood; **: *p* < 0.01; ***: *p* < 0.001.

**Table S2.** Sensitivity analysis

| **Exposure** | **Case/Total** | **Prevalence**  **(%)** | **APR^#^ (95% CI)** | **Full term birth** | | | **Preterm birth** | | |
| --- | --- | --- | --- | --- | --- | --- | --- | --- | --- |
|  |  |  |  | **Case/Total** | **Prevalence**  **(%)** | **APR^#^ (95% CI)** | **Case/Total** | **Prevalence**  **(%)** | **APR^#^ (95% CI)** |
| Screen exposure |  |  |  |  |  |  |  |  |  |
| No | 65/1972 | 3.30 | 1.00 | 61/1820 | 3.35 | 1.00 | 4/152 | 2.63 | 0.76 (0.28, 2.10) |
| Yes | 1957/26057 | 7.51 | 2.25 (1.76, 2.88)^***^ | 1759/24107 | 7.30 | 2.18 (1.69, 2.82)^***^ | 198/1950 | 10.15 | 3.06 (2.30, 4.08)^***^ |
| Initial age of exposure to screens | | | |  |  |  |  |  |  |
| No | 65/1972 | 3.30 | 1.00 | 61/1820 | 3.35 | 1.00 | 4/152 | 2.63 | 0.76 (0.28, 2.09) |
| 0 to 1 year old | 1032/10198 | 10.12 | 3.10 (2.41, 3.98)^***^ | 923/9405 | 9.81 | 3.01 (2.32, 3.90)^***^ | 109/793 | 13.75 | 4.23 (3.09, 5.80)^***^ |
| 1 to 2 years old | 447/6378 | 7.01 | 2.13 (1.64, 2.77)^***^ | 401/5903 | 6.79 | 2.05 (1.56, 2.69)^***^ | 46/475 | 9.68 | 2.96 (2.02, 4.34)^***^ |
| 2 to 3 years old | 265/4556 | 5.82 | 1.73 (1.32, 2.28)^***^ | 241/4227 | 5.70 | 1.68 (1.27, 2.23)^***^ | 24/329 | 7.29 | 2.13 (1.33, 3.41)^**^ |
| After 3 years old | 213/4925 | 4.32 | 1.24 (0.94, 1.64) | 194/4572 | 4.24 | 1.19 (0.89, 1.59) | 19/353 | 5.38 | 1.52 (0.91, 2.54) |

APR: adjusted prevalence ratio; CI: confidence intervals; #: Adjusted for children’s age, gender, maternal age at childbirth, paternal age at childbirth, maternal education level, paternal educational level, monthly household income, preterm birth, birth weight and passive smoking during early childhood; **: *p* < 0.01; ***: *p* < 0.001.
