# Supplementary figures and images for: Screen Exposure during Early Life and the Increased Risk of Astigmatism among Preschool Children: Findings from Longhua Child Cohort Study
Source: Int J Environ Res Public Health. 2020 Mar 26;17(7):2216. doi: 10.3390/ijerph17072216 (PMC7177845; doi:10.3390/ijerph17072216)

**
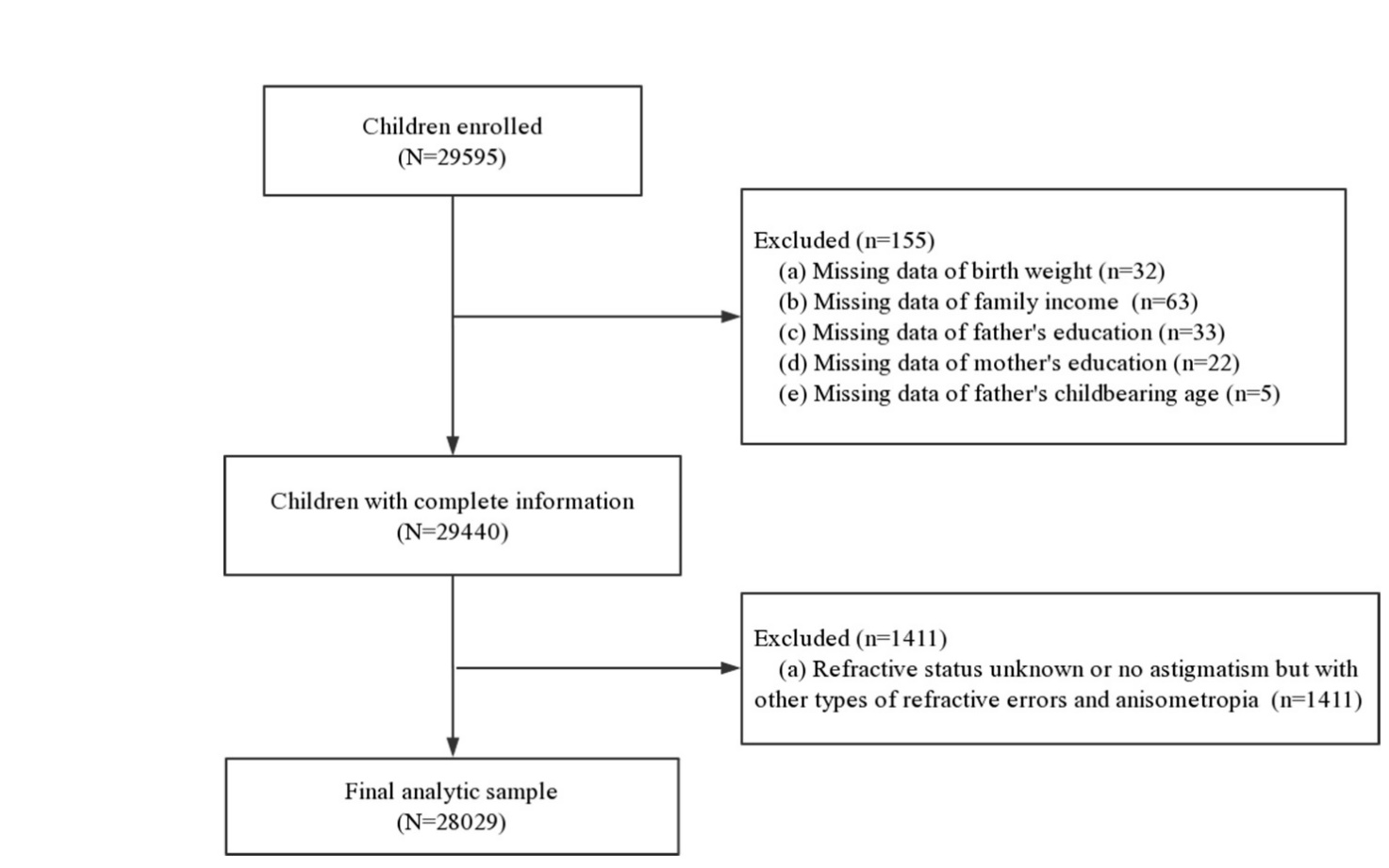
Figure S1.** The flow diagram of participant selection

Supplement: Supplementary file 1 [file ijerph-17-02216-s001.zip › ijerph-750615-supplementary/Figure S1_25_March_clean version.docx]
